# Influence of global gene regulatory networks on single cell heterogeneity of green fluorescent protein production in *Bacillus subtilis*

**DOI:** 10.1186/s12934-018-0985-9

**Published:** 2018-08-30

**Authors:** Haojie Cao, Oscar P. Kuipers

**Affiliations:** Department of Molecular Genetics, Groningen Biomolecular Sciences and Biotechnology Institute, University of Groningen, Groningen, The Netherlands

**Keywords:** *Bacillus subtilis*, Superfolder green fluorescent protein (sfGFP), Heterogeneous expression, Global transcriptional regulation, Production level, Single cell analysis, Phenotypic noise

## Abstract

**Background:**

Gram-positive bacterium *Bacillus subtilis* has been extensively studied as a microbial cell factory for high-level producing a wide range of interesting products. Green fluorescent protein (GFP) is commonly used as a marker for determining the strength of a given promoter or for the subcellular localization of a fusion protein. However, the inherent heterogeneity of GFP expression among individual cells that can arise from global regulation differences in the expression host, has not yet been systematically assessed. *B. subtilis* strains with single mutation(s) in the two major transcriptional regulators CcpA and/or CodY were earlier found to improve overall heterologous protein production levels. Here, we investigate the dynamic production performance of GFP in the reporter strains with chromosomally integrated P_*hyspank*_-sfGFP(Sp).

**Results:**

The mutation R214C in the DNA-binding domain of CodY effectively enhances GFP production at the population level relative to two other strains, i.e. wildtype (WT) and CcpA^T19S^. During the late stationary phase, the high- and low-level GFP-producing cells coexist in the WT population, while the CodY^R214C^ population at the single-cell level shows higher phenotypic homogeneity of fluorescence signals.

**Conclusion:**

Expression of GFP is prominently heterogeneous in the WT *B. subtilis* cells, and this phenotypic heterogeneity can be significantly reduced by CodY^R214C^ mutation. The rates of production heterogeneity show a high correlation to the overall GFP yields. Moreover, the toolkit of flow cytometry and fluorescence microscopy that can achieve real-time profiles of GFP production performance in various strains may facilitate the further use of *B. subtilis* as a cell factory.

## Background

The gradual but very rapid accumulation of genetic information and the fast development of experimental approaches have opened up many new frontiers in the cellular investigation [1]. The traditional bulk-scale measurements that only investigate the average values for a population of cells give an incomplete picture of what happens in bacterial cultures. The information on individual cells is needed for correctly monitoring biological processes. It has become evident that various subpopulations of bacteria can exist under certain conditions, with cells in distinct physiological or developmental states [2, 3]. Multiple studies have been focused on the development and utilization of single-cell techniques, which aid the research on the cellular behavior of individual cells in bacterial populations [4, 5].

It is widely recognized that bacterial cells with the same genetic information (clonal populations) can display a multitude of distinct phenotypes, even when exposed to the same environment; this phenomenon is known as phenotypic heterogeneity [6]. *Bacillus subtilis*, the best-characterized member of low-GC Gram-positive bacterial species, has been studied extensively with respect to phenotypic diversity. When nutrients are limited, *B. subtilis* in the stationary phase generates a mixed population, in which some cells form spores that are highly resistant to external stresses [7]. Additionally, a subset of cells that have entered into the sporulation state can secrete an extracellular ‘killing factor’ and toxin to block sister cells from sporulating and to stimulate the lysis of them [8]. In certain conditions, a subpopulation of the *B. subtilis* cells can enter into the competent state, enabling them to take up DNA from the environment [9, 10]. Heterogeneity also plays an important role in biofilm formation, which results in a subpopulation generating extracellular matrix material that tightly holds the surrounding cells together to form a robust biofilm [11]. Moreover, during exponential growth, a fraction of cells manage to express *sigD*, which is necessary for flagellar production, resulting in the cells to be motile [2].

Phenotypic heterogeneity, which mostly results from heterogeneous gene expression, increases the survival chance of a subpopulation that is better adapted to changing conditions [12–15]. There are three main factors that control the dynamic cellular behavior: (i) the circuit architecture or regulatory interaction patterns; (ii) quantitative parameters, such as promoter strengths; and (iii) stochastic fluctuations or “noise”, which depends on the availability of certain cellular components [16]. In general, the noise of gene expression arises from two sources. The “intrinsic” noise is generated by the inherent stochasticity of biochemical processes such as transcription and translation, causing a particular gene to be expressed at different levels in the cells at precisely the same state. On the other hand, the fluctuations in the states or accumulations of crucial cellular components such as regulatory proteins and polymerases represent “extrinsic” noise, leading indirectly to particular gene expression variation and which has a global effect [4, 17].

A wide variety of proteins have been chosen as reporters for benchmarking gene expression in order to study the mechanisms of phenotypic heterogeneity. In *B. subtilis*, the mostly used reporters include *lacZ*, encoding the β-galactosidase from *E. coli* [18], *luxAB*, encoding the luciferase from *Vibrio harveyi* [19], *mCherry*, encoding an enhanced red fluorescent protein from *Discosoma* sp. [20] and *gfp*, encoding the green fluorescent protein (GFP) from *Aequorea victoria* [21]. GFP and its derivatives have been extensively utilized in the study of protein localization or promoter activity in living cells [22], which has tremendously increased our knowledge of bacterial cell biology [23–25]. These analyses can be carried out using flow cytometry, fluorescence microscopy or both [26, 27]. Flow cytometry facilitates the rapid analysis of cells in the population, while time-lapse microscopy follows the behavior of individual cells over time and dynamic movements of proteins within a single cell [28–31]. A previous study from our laboratory benchmarked the expression of a library of GFP variants in three model microorganisms, i.e. *B. subtilis, Streptococcus pneumoniae,* and *Lactococcus lactis* [32]. Surprisingly, the superfolder GFP with codon optimization specifically for *S. pneumoniae*–sfGFP(Sp) displayed the highest fluorescence intensity and relatively low phenotypic noise in *B. subtilis.*

In *B. subtilis*, the pleiotropic transcriptional regulators CcpA and CodY behave either as a repressor or activator of gene expression by specifically binding to a sequence located in or near the promoter region of target genes. Therefore, these two regulatory proteins provide a top layer of metabolic networks by regulating genes that are involved in the carbon overflow, and citric acid cycle pathways, BCAA biosynthetic pathway, and the interplay between carbon and nitrogen metabolism [33]. In an earlier study, we explored the heterologous protein production potential of *B. subtilis* by genetically altering the two global regulators, which demonstrated that amino acid substitutions among the DNA-binding regions [34]. The mutations CodY^R214C^ and CcpA^T19S^ in one cell resulted in the reorganization of metabolic networks, which eventually improved the intracellular synthesis of β-galactosidase (*P*_*hyspank*_-*lacZ*) and other soluble proteins. In the present research, the robustly folded version of GFP–sfGFP(Sp) was utilized as a reporter protein to quantify the productivity of the wildtype and the obtained mutant CodY^R214C^CcpA^T19S^ over time, both at the population and single-cell level. Notably, this investigation points to altered production levels of GFP and great variation between single cells, depending on the central regulatory metabolic pathways operating in the WT and mutant cells.

## Results and discussion

### The alteration of global regulatory networks significantly impacts the GFP production in *B. subtilis*

As presented previously, we selected out desired phenotypes with higher product yields of the reporter protein (β-galactosidase) by consecutively screening the randomly mutagenized libraries of CodY and CcpA [34]. The best mutant strain CodY^R214C^CcpA^T19S^ that contains crucial mutations within the DNA-binding HTH motifs, shows significantly reprogrammed central carbon and nitrogen metabolic pathways, and this overall metabolic shift leads to a twofold increase of β-galactosidase production (*P*_*hyspank*_-*lacZ*) in comparison to the WT [34]. To investigate the expression of another classic reporter, GFP, in the genetically modified expression hosts, the sfGFP(Sp) was utilized in this research. Moreover, since the plasmid-based expression systems can cause additional heterogeneity due to copy number variation and polar fixation effects [35, 36], we integrated the expression cassette P_*hyspank*_-sfGFP(Sp) into the *amyE* locus in *B. subtilis* 168 WT, CodY^R214C^, CcpA^T19S^, CodY^R214C^CcpA^T19S^ to obtain the four reporter strains.

**Fig. 1:**
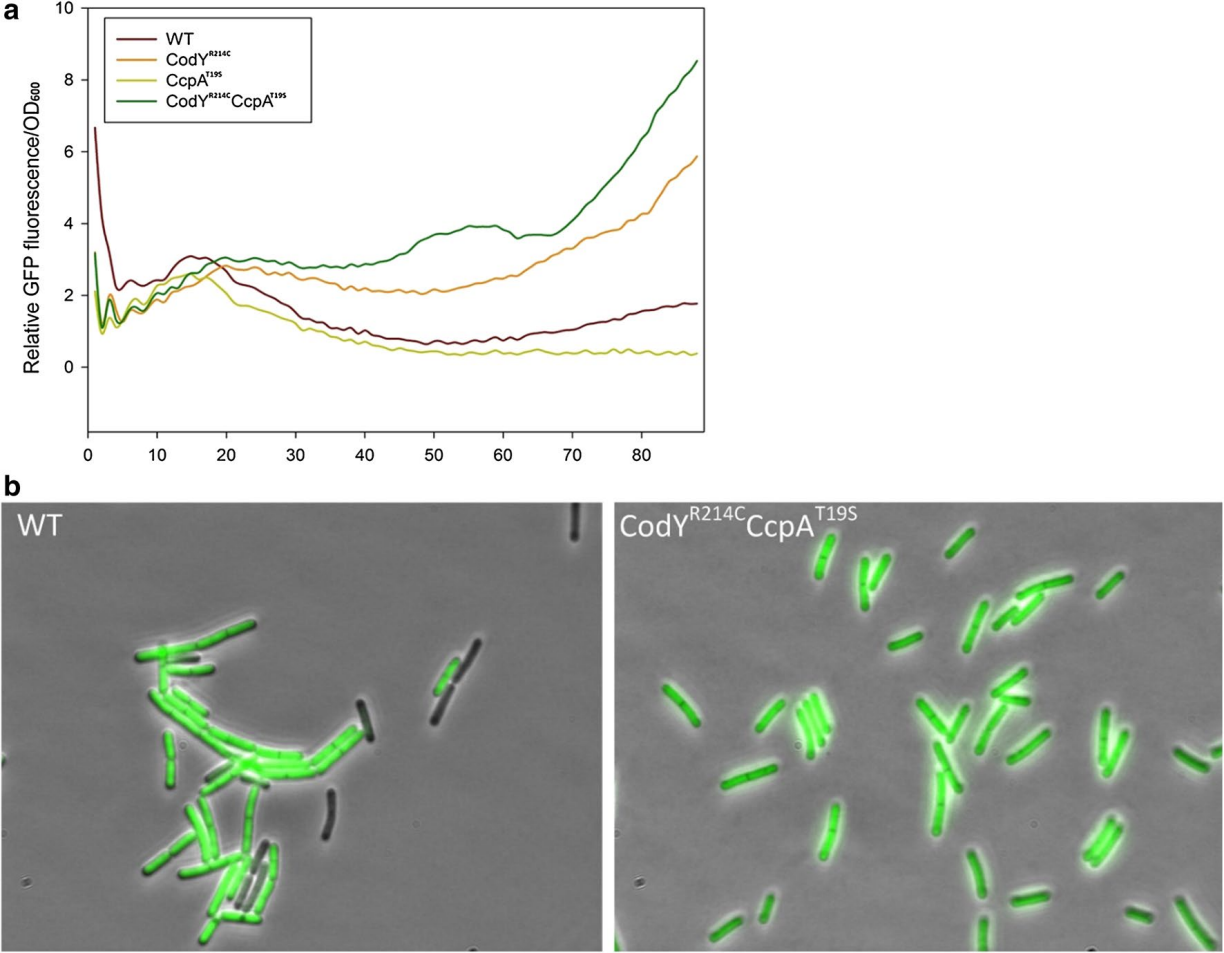
**a** Fluorescence intensity/OD_600_ of various *B. subtilis* strains in microtiter plates. Strains were grown in LB supplemented with 1.0% glucose and 0.1 mM IPTG under the same culture condition (37 °C, 220 rpm). Fluorescence intensity and OD_600_ were recorded by microplate reader every 15 min, the numbers on the x-axis represent the time points. We calculated the relative value of GFP expression level by using the formula: GFP fluorescence intensity/OD_600_. Experiments were performed in triplicate, but for clarity, only one representative line of the mean value is shown. **b** Visualization of green fluorescent protein production in *B. subtilis* by fluorescence microscopy. The overnight pre-culture was diluted to OD_600_ of 0.1 in fresh production medium (LB, 1.0% glucose, 0.1 mM IPTG). Subsequently, the mixture was incubated in flasks at 37 °C, 220 rpm for 5 h, and then the culture was immediately taken for fluorescence microscopy

Subsequently, we grew all the strains and induced the GFP expression identically in microtiter plates, and the fluorescence and growth were monitored using a plate reader (VarioskanLUX, Thermo Fisher) over time. As shown in Fig. 1a, during the 22 h’s incubation, the host CodY^R214C^ and CodY^R214C^CcpA^T19S^ produced higher levels of GFP, while the WT and CcpA^T19S^ generated relatively lower amounts of GFP under identical culture conditions. Since only a rough estimation of the fluorescence intensity at the population level can be determined in the microtiter plate reader, and the corresponding fluorescence signals were getting variable after 5 h, the cultures of CodY^R214C^CcpA^T19S^ and WT at that time point were subjected to fluorescence microscopy for visualizing and comparing the GFP expression at the single-cell level. As illustrated in Fig. 1b, there was a clear fluorescence signal variation among the WT cells, which demonstrated that the expression of the sfGFP in *B. subtilis* 168 is heterogeneous. In comparison, the fluorescence signals of individual CodY^R214C^CcpA^T19S^ cells were more homogeneous (Fig. 1b). Taken together, the overall GFP production was different in individual cells of the *B. subtilis* strains when various versions of CodY and/or CcpA were used. Compared with the WT control, the hosts containing the mutation CodY^R214C^ could significantly increase green fluorescent protein production, as was the case for β-galactosidase production [34]. Notably, the superfolder GFP was most heterogeneously expressed in WT cells.

### The rewired central nitrogen metabolism plays a crucial role in the GFP production enhancement

**Fig. 2:**
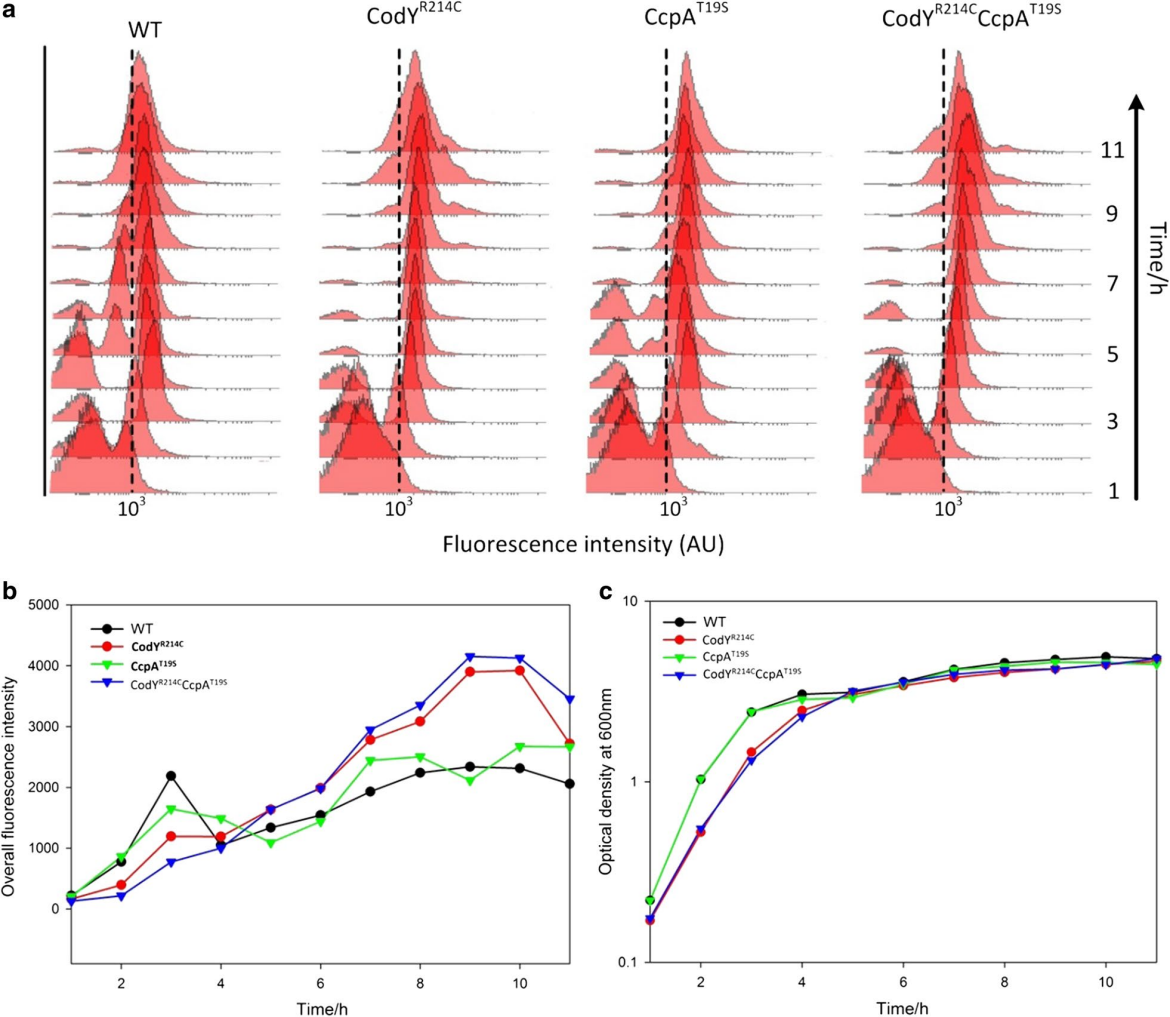
The expression of sfGFP(Sp) in various *B. subtilis* strains. *B. subtilis* WT, CodY^R214C^, CcpA^T19S^, CodY^R214C^CcpA^T19S^ harboring *amyE*::P_*hyspank*_-*sfgfp(Sp)* were grown in flasks with LB supplemented with 1.0% glucose and 0.1 mM IPTG under the same growth conditions (37 °C, 220 rpm). Samples were harvested for both fluorescence and OD_600_ measurement per hour. **a** Flow cytometric analysis of GFP expression. Dotted lines were placed at 10^3^ Arbitrary Units (AU) to serve as a reference of the fluorescence distributions. **b** The mean fluorescence intensity of the whole population over time. **c** The optical density at 600 nm of various strains was measured by spectrophotometry. For WT, CcpA^T19S^, the exponential phase is 1–4 h, and start the stationary phase from the point of 4 h; for CodY^R214C^ and CodY^R214C^CcpA^T19S^, the exponential phase is 1–5 h, and enter the stationary phase from the time point of 5 h

To reveal the mechanism behind the upshift of GFP production and to elucidate cellular behavior during expression, fluorescence microscopy and flow cytometric analysis of GFP production in the four strains (168, CodY^R214C^, CcpA^T19S^, CodY^R214C^CcpA^T19S^) were performed in parallel. Figure 2a shows the flow cytometry tracings of the four mutants when cultured under the same conditions. The corresponding mean fluorescence intensity and optical density for each time point are presented in Fig. 2b, c respectively. In line with the prior observation, the CodY^R214C^ and CodY^R214C^CcpA^T19S^ showed higher GFP signals than the other strains at the population level. The WT and CcpA^T19S^ exhibited similar curves to each other concerning the growth and the fluorescence intensity, being significantly different from that of CodY^R214C^ and CodY^R214C^CcpA^T19S^, which showed similar growth and GFP production to each other. WT and CcpA^T19S^ reached stationary phase 1 h earlier than the two strains containing CodY^R214C^ (Fig. 2c). The GFP production level in the latter two hosts, especially during the stationary phase, was higher than that of the former two (Fig. 2b). Furthermore, there was a detectable decline of mean fluorescence intensity in 50,000 cells of WT and CcpA^T19S^ after the first 3 h gradual rise. In contrast, the accumulation of GFP in CodY^R214C^ and CodY^R214C^CcpA^T19S^ improved continuously until the late stationary phase. In summary, the amino acid substitution R214C in CodY cause a stronger GFP synthesis ability at a slight expense of growth rate, while the mutation CcpA^T19S^ did not play a positive role in the expression of the reporter protein–sfGFP(Sp) in *B. subtilis*.

### Phenotypic noise, related to global regulation, negatively correlates to the overall GFP production level

**Fig. 3:**
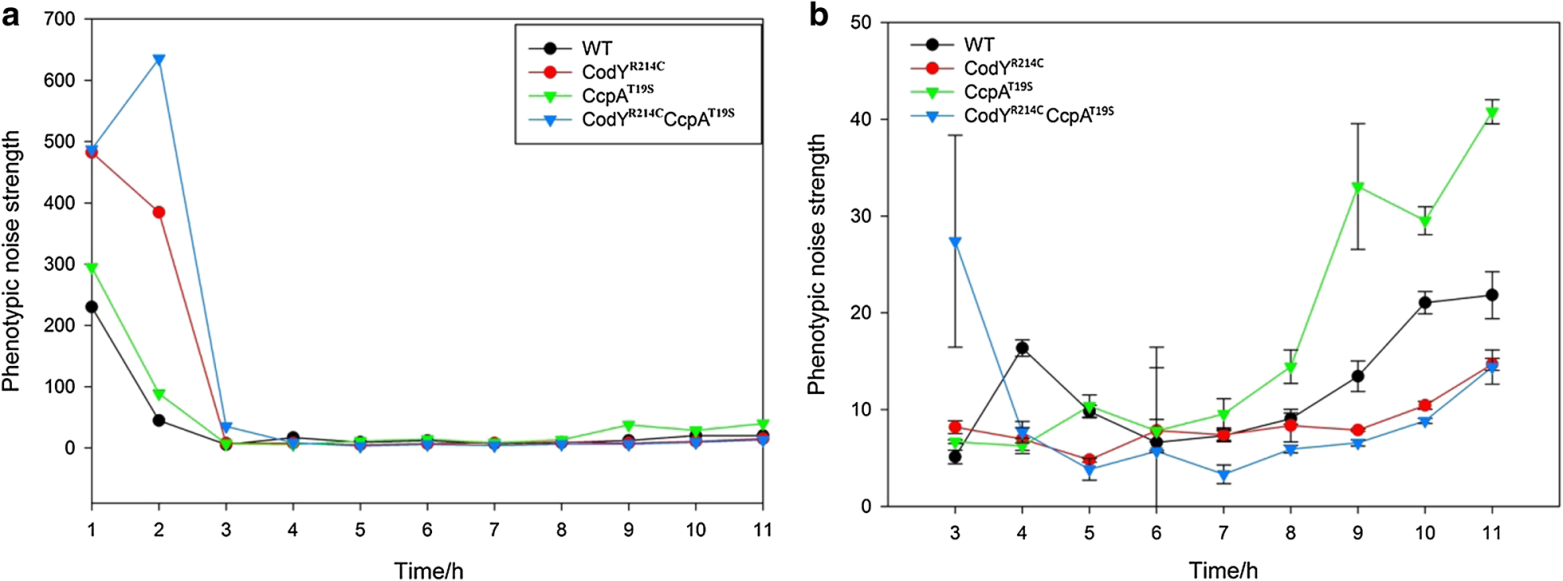
The phenotypic noise of GFP expression in various hosts. The phenotypic noise was calculated by using the formula: σ_p_^2^/〈p〉 (variance/mean), σ_p_ was also named the coefficient of variation (CV) in the flow cytometric analysis. All the experiments were performed in triplicate, but for clarity, only the average lines of whole 11 h are shown in **a**, while the average lines with error bars from 3 to 11 h are presented in **b**

The distribution of the expression of a single gene can be defined by the mean value of expression level indicated by 〈*p*〉 with a standard deviation-σ_*p*_ or coefficient of variation (CV) [37]. The phenotypic noise strength (σ_*p*_/〈*p*〉), is extensively applied for the measure of noise [1, 15, 38]. Based on the data from the flow cytometric analysis, we quantified the spread of GFP fluorescence signals in a population of various strains. Since the different versions of the regulator(s) in the expression hosts are the only variable during the GFP synthesis process, the extrinsic noise that arises from the regulation, should play a crucial role in the final GFP yield. As shown in Fig. 3a, the noise strength of the GFP expression in *B. subtilis* is dynamic over time. Overall, the phenotypic noise was high at the beginning of growth and then dropped sharply in the following 4 h (Fig. 3a). This is probably due to the IPTG induction, which controls the GFP production, that does not start simultaneously in all cells [39]. After remaining at a steady state for an extended period, the noise increased again when cultures reached late stationary phase (Fig. 3b). In addition, a significant difference with regard to phenotypic noise was observed from the four assessed strains after 8 h of growth. The CcpA^T19S^ strain showed the strongest noise value of GFP expression compared to the other three hosts, and the CodY^R214C^CcpA^T19S^ strain showed the lowest noise among all the expression hosts. We thus conclude that the strength of noise is opposed to the corresponding mean fluorescence intensity in various strains. This indicates that the different versions of global regulators cause diverse extrinsic noise levels during the overexpression of sfGFP(Sp), which eventually results in different levels of the overall GFP yield. This observation is consistent with a previous conclusion that the overall yields of reporter protein can be enhanced by decreasing the expression noise in *B. subtilis* [40].

### Characterization of GFP production at the single-cell level

**Fig. 4:**
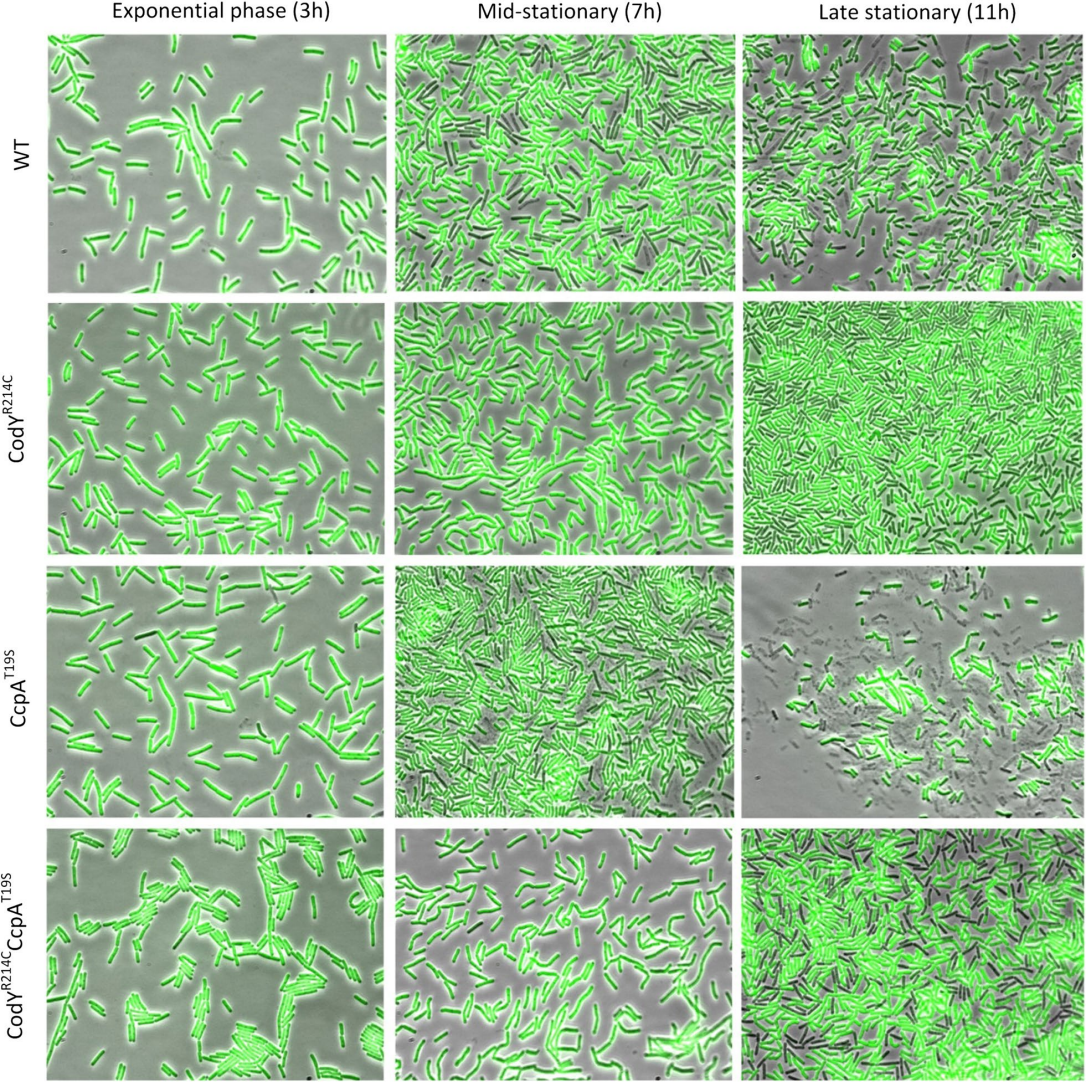
Phenotypic heterogeneity of various strains during growth. The strains were grown at 37 °C, 220 rpm in LB supplemented with 1.0% glucose and 0.1 mM IPTG for 11 h. The GFP fluorescence images and phase contrast images of cells at different time points were acquired, and the merged micrographs are presented

Fluorescence microscopy was carried out to visualize the production of sfGFP(Sp) in single cells per hour. Here, we picked three representative images of the cells in exponential, mid-stationary, and late stationary phase for further analysis. As indicated in Fig. 4, during the exponential phase, all the cells of the four detected strains show strong signal and similarity in the fluorescence intensity. When the cultures reached the stationary phase, most cellular heterogeneity with respect to fluorescence occurred in WT and CcpA^T19S^. This phenotypic diversity is most prominent during mid-stationary growth after 7 h. Dark cells with low GFP activity co-exist with the cells having strong GFP intensity in the cultures of the above two strains. During the mid-stationary growth phase, cellular heterogeneity of CodY^R214C^ and CodY^R214C^CcpA^T19S^ was hardly visible. Finally, the GFP is expressed heterogeneously in the strains with CodY^R214C^ in the late stationary phase, while the cells of the other two strains, especially the CcpA^T19S^, already lysed severely. This is consistent with the observation in Fig. 2b, the GFP intensity in CodY^R214C^ and CodY^R214C^CcpA^T19S^ reduced at the end of 11 h’ expression. This reflects that the activity of cellular processes decreased, owing to the short supply of essential nutrient sources when the strains entered into the late stationary phase. During the same growth phase, some of the dark cells of CcpA^T19S^ lysed while some bight cells become dark due to the decreasing amount of available nutrients. The slight time difference between these two processes may result in the fluctuation of the overall GFP production level occurred for the strain CcpA^T19S^ (Fig. 2b).

### Characterization of GFP production at the subpopulation level

**Fig. 5:**
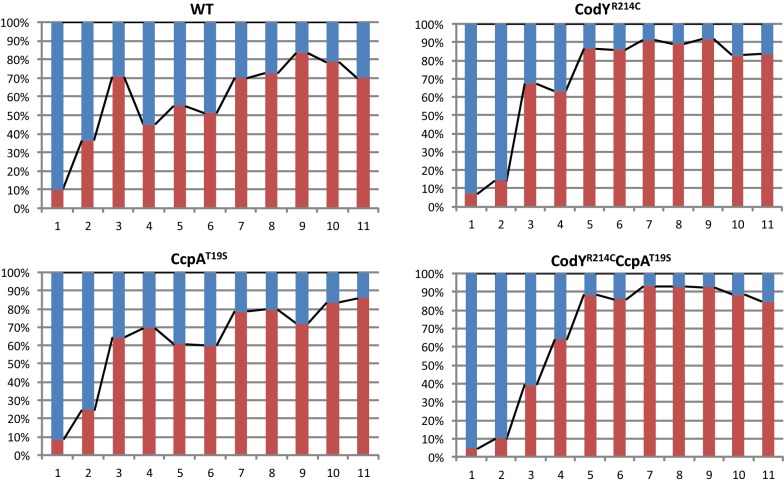
The dynamic proportion of the two GFP intensity subpopulations. The red bars represent positive subpopulations (> 10^3^ AU), and the blue histograms represent negative subpopulations (< 10^3^ AU). The numbers on the x-axis represent the time points (hour)

To further study GFP production in subpopulations, we analyzed the flow cytometry results of different strains by Flowing Software. We set the fluorescence intensity 10^3^ AU as the cutoff value and defined the subpopulations as negative (< 10^3^ AU) or positive (> 10^3^ AU). As displayed in Fig. 5, the two strains harboring the WT version of CodY showed similarity in the percentage of the two subpopulations, while the two hosts carrying CodY^R214C^ also shared similar subpopulation proportions. During the stationary growth phase, the overall percentages of positive subpopulations for the CodY^R214C^ and CodY^R214C^CcpA^T19S^ strains were obviously higher than that of the WT and CcpA^T19S^. If we combine Figs. 2 and 5, it is interesting to note that the positive percentages show high consistency with GFP expression performance in expression hosts harboring various versions of CodY and/or CcpA. The overall fluorescence signal strength depends on the positive subpopulations in various strains.

### Metabolic burden might affect the heterologous expression of GFP

Metabolic burden, a known phenomenon for heterologous expression systems, is caused by the fact that the overexpression pathways of foreign proteins can take up a large proportion of the nutrient source fluxes, which then influences the original metabolic distribution in the cell, and cause serious physiological problems and finally results in lower yields of target products [41–43]. In a previous study, we reprogrammed the metabolic regulatory networks, and found that a more strongly repressed carbon metabolism and de-repressed nitrogen metabolism coordinately contribute to an increase of the reporter protein β-galactosidase production in *B. subtilis* [34]. The production improvements were found to be consistent with upregulation of several nitrogen metabolic operons, and this was regarded to reduce the metabolic burden of β-gal overexpression in the genetically modified strains. The balanced and modified metabolic networks with increased uptake and utilization ability of arginine, ornithine, citrulline, and histidine could also weaken the extrinsic noise of GFP expression in the CodY^R214C^CcpA^T19S^. Different from the previous observation, strain CcpA^T19S^ does not have an advantage in the expression of sfGFP(Sp), which is slightly lower than the WT control. This is in accordance with the fact that protein production improvement is performed in a protein-specific way [44]. Nevertheless, based on population-scale analysis, the mutation CcpA^T19S^ can still further improve the GFP expression on the basis of the improvement in CodY^R214C^. This shows that the effects of mutation CodY^R214C^ and CcpA^T19S^ on the final production of sfGFP(Sp) are more complex than simple addition. To sum up, the CodY^R214C^CcpA^T19S^ strain displays balanced metabolic flux distributions between essential cellular processes and heterologous over-expression pathway probably has a lower metabolic burden. This not only increased the overall product yield but also decreased the phenotypic heterogeneity of sfGFP(Sp) expression in *B. subtilis*, a property generally useful for overproduction of any soluble intracellular protein.

## Conclusion

In this study, we investigated the production of sfGFP(Sp) in strains with mutation(s) in CodY and/or CcpA and the WT strain as the control. We demonstrated that the mutation CodY^R214C^ improves the overall expression of reporter protein sfGFP(Sp) significantly, with a slight decrease of the growth rate, while the CcpA^T19S^ mutant slightly reduces the GFP synthesis. Nevertheless, when the two amino acid substitutions among the DNA-binding HTH motif of CodY and CcpA were combined, this yielded the best GFP producer—CodY^R214C^CcpA^T19S^. Furthermore, the phenotypic noise clearly differs between different mutants of the global regulator(s). This extrinsic noise comes from global regulation and is shown to be negatively correlated with GFP production in our cell factories. In addition, the single-cell and subpopulation analyses demonstrated that the cells of WT and CcpA^T19S^ show stronger heterogeneity during the expression process over time. Although the full understanding of the mechanisms underlying expression heterogeneity is still incomplete, this study provides novel insights into decreasing cellular diversity and directs the way to further increase heterologous protein production in cell factories.

## Methods

### Plasmids, bacterial strains, and medium

The plasmids and bacterial strains used in this study are listed in Table 1. All the *B. subtilis* and *E. coli* were grown at 37 °C with shaking (220 rpm) in liquid Lysogeny Broth (LB) unless otherwise indicated, and 1.0% glucose was supplemented in the media for all the *B. subtilis* strains. For solid medium, 1.5% (wt/vol) agar was added to the LB. Antibiotics were added when necessary as follows: 100 mg/ml ampicillin for *E. coli*, 5 mg/ml kanamycin and chloramphenicol, 100 mg/ml spectinomycin for *B. subtilis*. When required, 0.1 mM IPTG (isopropyl-β-d-thiogalactosidase) was added to the medium for activation of the IPTG-inducible expression system.

**Table 1:** The plasmids and bacterial strains used in this study

Strains and plasmids	Phenotype or property	Source or references
Strains		
*B. subtilis*		
168	*trpC2*	[47]
168_sfGFP(Sp)	*trpC2, amyE::P*_*hyspank*_-*sfgfp(Sp) spc*^*r*^	[32]
168_sfGFP(Sp)_CodY^R214C^	*trpC2, codY*^*R214C*^ *cm*^*r*^*, amyE::P*_*hyspank*_-*sfgfp(Sp) spc*^*r*^	This study
168_sfGFP(Sp)_CcpA^T19S^	*trpC2, ccpA*^*T19S*^ *km*^*r*^*, amyE::P*_*hyspank*_-*sfgfp(Sp) spc*^*r*^	This study
168_sfGFP(Sp)_CodY^R214C^CcpA^T19S^	*trpC2, codY*^*R214C*^ *cm*^*r*^*, ccpA*^*T19S*^ *km*^*r*^*, amyE::P*_*hyspank*_- *sfgfp(Sp) spc*^*r*^	This study
*E. coli*		
MC1061	F^−^, *araD*139, Δ(*ara*-*leu*)7696, Δ(*lac*)X74, *galU*, *galK, hsdR2, mcrA, mcrB1, rspL*	[48]
Plasmids		
pCH3_CcpA^T19S^	pUC18*_aroA_ccpA*^*T19S*^*_km*^*r*^*_ytxD*	[34]
pJV153	pUC18_*clpY_codY*^*R214C*^*_cm*^*r*^*_flgB*	[34]

### Recombinant DNA techniques and oligonucleotides

Procedures for DNA purification, restriction, ligation, gel electrophoresis and transformation of *E. coli* were carried out as previously described [45]. *B. subtilis* was naturally transformed as described before [46]. T4 DNA ligase, Fastdigest Restriction enzymes and DNA polymerases (Phusion and DreamTaq) were purchased from Thermo Fisher Scientific (Landsmeer, Netherlands). Chromosomal DNA of the *B. subtilis* 168 and the constructed plasmids in this research were used as templates for PCR. The NucleoSpin Plasmid EasyPure and Gel & PCR Clean-up kits were purchased from BIOKE (Leiden, Netherlands). All the reagents used were bought from Sigma unless otherwise indicated. Oligonucleotides were synthesized by Biolegio (Nijmegen, Netherlands). Sequencing of all our constructs was performed at MacroGen (Amsterdam, Netherlands).

### Construction of bacterial strains

*Bacillus subtilis* strain 168_sfGFP(Sp)_CodY^R214C^ was obtained by homologous double crossover recombination of plasmid pJV153 into the flanking region of *codY* in *B. subtilis* 168. Strain 168_sfGFP(Sp)_CcpA^T19S^ was obtained by the integration of plasmid pCH3_CcpA^T19S^ into the specific chromosomal region of *B. subtilis* 168. Transformants were selected on LB agar plates containing appropriate antibiotic(s), after overnight incubation at 37 °C. Correct integration was verified by PCR and sequence analysis. The strain 168_sfGFP(Sp)_CodY^R214C^CcpA^T19S^ was constructed in the same way as described above.

### Microplate experiments

Single colonies of required strains were picked from LB agar plates with antibiotics and were incubated at 37 °C, 220 rpm overnight. The day after, the O/N cultures were diluted in a 96-well microtiter plate to OD_600_-0.1 with 200 µl fresh LB medium containing 1.0% glucose and 0.1 mM ITPG. Plates were incubated at 37 °C and 220 rpm shaking in the plate reader-VarioskanLUX (Thermo Fisher) with a GFP filter set (excitation at 485/20 nm, emission 535/25), and the absorbance was measured at 600 nm. The values of GFP intensity and OD_600_ were automatically recorded every 15 min for 22 h, data of all samples were collected in triplicates. All the optical density and fluorescence values were corrected for the background of the medium by the following formula: (GFP_reporter _− GFP_medium_)/(OD_reporter _− OD_medium_) [49].

### Flow cytometry

All the strains were streaked on LB agar plates supplemented with a specific antibiotic, and the single colonies were picked up and grown overnight in LB medium at 37 °C, 220 rpm. Next morning, the pre-cultures were diluted to OD_600_-0.1 in fresh LB supplemented with 1.0% glucose and 0.1 mM IPTG and further incubated in a 37 °C shaker. Subsequently, the cultures of each time point were prepared for flow cytometry as described before [3, 12]. Cells were diluted 10–20 times in phosphate buffered saline (PBS) and directly measured on the Becton–Dickinson FACSCanto (BD BioSciences, USA) with an Argon laser (488 nm). For each sample, the green fluorescent signals of 50,000 cells were collected by a FITC filter. The fluorescent intensity was calculated in Arbitrary Units (AU). All the captured data was further analyzed using Flowing Software (http://www.flowingsoftware.com/).

### Fluorescence microscopy

In parallel, the above-described cultures of each time point were also prepared for fluorescence microscopy and applied to agarose slides as described before [25]. The expression of the fluorescent protein was analyzed by fluorescence microscopy (Nikon Eclipse Ti, Japan) equipped with a CoolsnapHQ2 CCD camera. Fluorescent signals from cells were visualized using 450–490 nm excitation and 500–550 nm emission for GFP fluorescence channel and an Intensilight light as phase contrast channel. Software NIS-Elements AR [50] was used for image capturing by 0.2 s exposure, and the final images for publication were generated by ImageJ software [51].
